# Research in Action—Students’ Perspectives on the Integration of Research Activities in Undergraduate Biomedical Curricula

**DOI:** 10.1007/s40670-021-01228-8

**Published:** 2021-02-18

**Authors:** Femmie de Vegt, Johannes D. M. Otten, Diederik R. H. de Bruijn, Helma Pluk, Iris A. L. M. van Rooij, Thom F. Oostendorp

**Affiliations:** 1 Department for Health Evidence, Radboud University Medical Center, Nijmegen, The Netherlands; 2 Department of Human Genetics, Radboud University Medical Center, Nijmegen, The Netherlands; 3 Department of Biochemistry, Radboud University Medical Center, Nijmegen, The Netherlands; 4 Donders Centre for Neuroscience, Radboud University Medical Center, Nijmegen, The Netherlands

**Keywords:** Academic skills, Research methods, Undergraduate curricula, Biomedical sciences

## Abstract

We describe and evaluate our practice-based learning approach for research in undergraduate students studying Biomedical Sciences at Radboud University Nijmegen, the Netherlands. First-year students who started their study between 2015 and 2018 actively participated in data collection and measurements, including anthropometry, electrocardiogram findings, genetic variants, and lifestyle habits. All data were entered into one anonymous database*,* which was used by students to analyze their research questions. In 2019, 44 of the 87 students (50%) valued active measurements better than questionnaires. Most students (strongly) agreed that they have learned about data collection and were inspired to learn more about biomedical research.

## Background

In recent years, there is a trend towards a more active involvement of students in research activities in (bio)medical curricula. A closer connection between research and teaching may help students to develop their academic skills, such as the ability to think critically, to analyze problems, and to report the results in a clear and concise way. These skills are necessary for their future career in a complex knowledge society [1–3]. There is also an urgency for a more active teaching approach, as active engagement confers a deeper understanding of science when students actively handle with research questions and methods than when they passively listen to answers [4].

Despite the general agreement regarding the relevance of active learning and research integration in (bio)medical curricula, there is no consensus on the best way how to do this in undergraduate curricula.

A well-known model between research and teaching, described by Healey, distinguishes two dimensions: one dimension ranges from “student as audience” to “student as participant” and the other dimension ranges from “emphasis on research content” to “emphasis on research processes and problems” [5]. In higher education, a major part of scientific education is in the more passive dimensions, with emphasis on content and students as audience. Recent data suggests that students will benefit from more active engagement in learning research skills. Making the student a participant in research activities will enhance learning outcomes [4, 6, 7].

In order to understand and appreciate biomedical research, it is important to integrate research early in (bio)medical education. Students develop research skills at a higher level if they are actively involved and participate in realistic research activities from the start of their academic education [3, 8, 9].

In this short paper, we describe and evaluate our practice-based learning approach in first-year students studying Biomedical Sciences at Radboud University Medical Center in Nijmegen, the Netherlands. The evaluation was based on students’ experiences and perspectives.

## Activity

### Student Research Database

*Research* is one of the longitudinal tracks in the Bachelor’s program Biomedical Sciences since the curriculum change of 2015. This track included several short courses in which students learn biomedical research methods, ranging from laboratory skills and performing physical measurements to data management and statistical analyses. Moreover, they learn to write a scientific paper and to present and (orally) communicate research findings.

In our practice-based learning approach, first-year students are actively involved in biomedical research by collecting data from themselves and their colleague-students. After obtaining informed consent, in the first quartile (10-week period), students measure and collect data concerning body weight, body circumferences, fat mass, and bone mass. In addition, students are asked to fill out the SQUASH questionnaire on physical activity [10, 11] and a short questionnaire on lifestyle habits. In the second quartile, they record ECGs to measure heart rate, heart rate variability, and other ECG properties (QRS, QT interval) in rest and during activity. In a laboratory class, students isolate DNA from saliva and use standard genetic lab techniques (PCR and Sanger sequencing) to measure four different genetic variants in their DNA. In addition, they measure four common traits that are known to be associated with these genotypes [12]. In the third quartile, students collect data on their usual food consumption by using a digital food diary for 3 days [13]. They also collect a morning-urine sample for biochemical analyses (urea, creatinine, pH, and dipstick).

Before the end of each quartile, all data collected are entered and merged into one student research database. In the first three quartiles, students also practice with data analyses and writing scientific reports concerning the specific data collection and measurements.

In quartile 4, during the course “Research Your Own Data,” all biomedical students get access to the (anonymous) student research database. They formulate a research question of their own choice, perform the statistical data analyses, and write a short research paper. The research papers are assessed using a rubric by experienced researchers who also give additional narrative feedback on the students’ research reports.

### Students Evaluation and Appreciation of Research Activities

Each year, around 100 students start with the Bachelor’s program Biomedical Sciences. In spring 2019, the first-year students who started their study in September 2018 and participated in the “Research” track in quartile 4 (*n* = 87) were asked for their experiences and appreciation of this practice-based learning approach. Students were asked to value the various measurements and questionnaires used for data collection (scale 1–10) and were asked for their reasons (not) to participate in the data collection and give suggestion for improvements. The answers on these open questions were independently coded by two of the authors (FdV and HO) and discussed to reach consensus.

The motivation and appreciation for research was evaluated by using an adapted version of the Student Perception of Research Integration Questionnaire (SPRIQ) (5-point Likert scale) [14]. We included eight questions about motivation and appreciation of bio medical research, participation in data collection and active engagement in biomedical research.

## Results

**Fig. 1:**
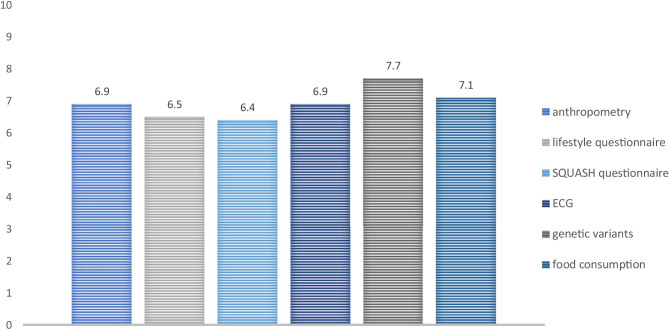
Value (grade 1–10) of students for data collection methods in the first year of Biomedical Sciences, Radboudumc Nijmegen

In April 2019, the *Student Research Database* included data from 298 individual students. Forty-four of the 87 invited first-year students (50%) filled out the evaluation and appreciation questionnaire in May 2019. Students valued the active measurements better than the questionnaires: the mean value for genetic variants was 7.7 (95% confidence interval (CI) 7.3–8.2) compared to 6.4 (95%CI 6.0–6.9) for the SQUASH physical activity questionnaire (Fig. 1). The participation rate in the data collection using questionnaires was also lower: 55% for the SQUASH questionnaire and 57% for the lifestyle questionnaire, compared to 91–100% for the anthropometry, ECG, and genetic variant measurements.

Most mentioned reasons for students to participate in the (voluntary) measurements and data collection were (1) “Necessary for assignment ‘Research your own data’” (*n* = 21, 28%), (2) “Interesting to know and measure own data” (*n* = 17, 23%), and (3) “To gain experience in research” (*n* = 12, 16%). There were only a few reasons mentioned why not to participate: “time and effort” (*n* = 5, 23%) and “instructions not clear” (*n* = 5, 23%). A few students mentioned privacy aspects (the anonymous dataset) as a reason to participate (*n* = 5, 5%), while a few others questioned the privacy (*n* = 3, 14%) and decided not to participate (Table 1).

**Table 1:** Reasons mentioned (not) to participate in the voluntary data collection in the first year of the Biomedical Sciences study—results from 44 students

	Number (%)
Reasons to participate	
Necessary for assignment “Research your own data”	21 (28)
Interesting to know and measure own data	17 (23)
To gain experience in research	12 (16)
Thought it was mandatory	8 (11)
Little effort	7 (10)
It was anonymous	5 (5)
Other	5 (7)
Reasons not to participate	
Time and effort	5 (23)
Instructions not clear	5 (23)
Forgotten	3 (14)
Privacy issue	3 (14)
Not scheduled in education activity	3 (14)
Other	3 (14)

**Fig. 2:**
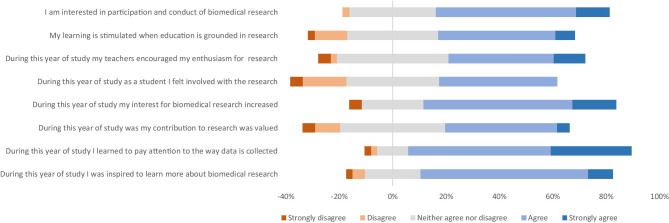
Motivation and appreciation for research by students using an adapted version of the Student Perception of Research Integration Questionnaire (SPRIQ) [14]

The answers on the adapted Student Perception of Research Integration Questionnaire (SPRIQ) are depicted in (Fig. 2) Seventy-two percent of the students (strongly) agreed that they were inspired to learn more about biomedical research. Eighty-four percent (strongly) agreed they have learned to pay attention to how data is collected and research is carried out. Forty-four percent of the student felt they were involved in research activities, whereas 21% felt not involved.

## Discussion

It is feasible and attractive to implement research skills in undergraduate programs by practice-based learning. When students gain experience in research by performing measurements and collecting data in their own group, they are more connected with the acquired data. Using these, more personal, data when performing statistical analyses and writing research reports actively introduced them in the world of biomedical research [15, 16].

Students seem to appreciate active measurements more than just filling out questionnaires, as is shown by the lower values and lower response rates for the questionnaires compared to other ways of data collection. However, the active measurements were scheduled in teaching activities, while the questionnaires were just sent by email, which may also have influenced these results.

The first-year biomedical students state that they have learned much about measurements and data collection, by being both a researcher and a participant, and they were inspired to learn more about biomedical research.

Ommering et al. endorse these results, as they plea to engage students in research activities from the beginning of their medical training, and also to challenge them by active participation in research projects [17]. However, the active involvement may be appreciated even more with an even closer connection between research and teaching. Therefore, a further expansion and integration of research in (bio)medical curricula is needed.
